# Electrode Architectures in Biophotoelectrochemical Systems for Sustainable Energy and Environmental Applications

**DOI:** 10.1002/cssc.71162

**Published:** 2026-10-29

**Authors:** Indra J. Budiarso, Masaki Umetsu, Yuta Nakayasu, Masanobu Kamitakahara

**Affiliations:** ^1^ Graduate School of Environmental Studies Tohoku University Sendai Miyagi Japan; ^2^ Frontier Research Institute for Interdisciplinary Sciences Tohoku University Sendai Miyagi Japan

**Keywords:** biocatalysis, CO_2_ reduction reaction, electrochemistry, energy recovery, hydrogen evolution, nitrogen fixation, photophysics

## Abstract

Current energy demand and the need for environmental stewardship call for transformative technologies that can simultaneously produce clean energy and remediate waste. Biophotoelectrochemical (BPEC) systems are promising platforms that couple the light‐harvesting capability of semiconductor photocatalysts with the catalytic precision of biocatalysts using an electrically addressable electrode. BPEC systems provide tunable reaction pathways, improved scalability, easier product handling, and flexibility in coupling various reactions by modifying the electrode architecture. This review presents a comprehensive discussion of these architectures in BPEC systems, beginning with the fundamental principles of how photoelectrodes and bioelectrodes work. This review then provides a systematic examination of two different electrode architectures: separated electrodes (photoelectrode | bioelectrode) and biohybrid photoelectrodes (biophotoelectrodes). The discussion covers their configurations, working principles, and representative applications such as H_2_ evolution, CO_2_ reduction, N_2_ fixation, pollutant removal, and energy recovery. The review also summarizes key performance metrics and current research trends. By integrating the complementary strengths of photoelectrodes and bioelectrodes, BPEC systems can overcome individual technological limitations and support sustainable energy and environmental applications through self‐biased operation and enhanced product selectivity. Finally, the review discusses the challenges and future directions of advancing BPEC systems into scalable, multifunctional platforms for next‐generation sustainable technologies.

## Graphical abstract

Biophotoelectrochemical system merges photoelectrochemical with bioelectrochemical system to enable solar‐driven redox reactions using microorganisms or enzymes. This approach offers multifunctional abilities such as selective H_2_ production, CO_2_ reduction, N_2_ fixation, pollutant removal, and energy recovery, highlighting new pathways for sustainable energy and environmental technologies.

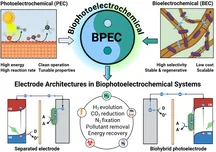

## 1 Introduction

Continued reliance on fossil fuels, coupled with population growth, could potentially cause a severe energy shortage in the future [1, 2], while the projected global population of 10.3 billion by the mid‐2080s will inevitably increase the volume of complex waste streams containing organic contaminants, pharmaceuticals, heavy metals, and microplastics. This poses a significant threat to environmental security [3, 4, 5, 6], making technologies that can address both energy generation and environmental remediation highly desirable [7, 8, 9, 10].

Solar energy utilization is of high interest because the solar irradiance is approximately 1360 W m^−2^ [11]. Although photovoltaic (PV) technologies have achieved commercial solar conversion efficiencies near 20% [12], their output is restricted to electricity. This limits their direct integration with chemical transformation and waste treatment processes [13]. Photoelectrochemical (PEC) systems, which were first demonstrated for water splitting [14], overcome this limitation. They directly convert solar energy into storable chemical fuels using photocatalytic materials [15, 16]. PEC systems offer strong redox driving forces and fast reaction kinetics, enabling solar‐driven applications such as fuel generation [17] and pollutant removal [18]. However, PEC systems often suffer from limited product selectivity and photostability under operating conditions [19, 20, 21, 22]. Conversely, bioelectrochemical (BEC) systems offer a different approach, using biocatalysts (whole cell and enzyme) to convert the chemical energy of pollutants into electricity or valuable products under mild conditions [23, 24, 25, 26]. Their high selectivity, stability, and scalability make BEC systems attractive for wastewater treatment and energy recovery [27]. However, biological constraints, such as slow extracellular electron transfer (EET) and limited metabolic rates, restrict reaction kinetics and energy output, hindering broader application [28].

The complementary strengths and limitations of PEC and BEC systems led to the development of biophotoelectrochemical (BPEC) systems. These combine PEC and BEC technologies, merging photogenerated redox power with biological selectivity. The goal is to achieve highly efficient, yet selective, redox reactions using light as the main energy source [29]. By integrating artificial materials into the machinery of biological systems, a unique approach is achieved. This approach addresses urgent energy and environmental challenges [29, 32]. Over the past 15 years, BPEC systems have demonstrated the capacity to facilitate several processes in an environmentally friendly manner, including the hydrogen evolution reaction (HER) [33], CO_2_ reduction reaction (CO_2_RR) [34], N_2_ fixation [35], pollutant removal [36], energy recovery [37], and even multiple functions simultaneously [38]. Previous reviews have classified BPEC systems broadly into three categories: suspension‐based, carrier‐supported, and electrode‐based, as shown in Figure 1 [29, 38]. However, since the term “electro” in “biophotoelectrochemical” implies the use of an electrically addressable electrode, this review reserves the term BPEC specifically for systems containing at least one such electrode. In this review, suspension‐based and carrier‐supported systems, which have sometimes been included within the broader scope of BPEC [29, 38], are referred to as “BPEC‐related systems”. Suspension‐based systems consist of dispersed photocatalyst powder added to a microbial growth medium [39] or attached to the surface of microorganisms [40]. Early suspension‐based BPEC‐related systems demonstrated feasibility; however, they were limited by mediator toxicity, light attenuation, the necessity of a sacrificial electron donor, and challenges associated with scaling up [41, 44]. Carrier‐supported BPEC‐related systems are composed of immobilized photocatalysts and biocatalysts on the surface of a porous carrier rather than floating freely in a solution [45]. Despite improved pollutant removal through sequential photocatalytic oxidation and biodegradation, these systems remain constrained by mass transfer, the necessity of a sacrificial electron donor, and light attenuation [44, 45].

**FIGURE 1:**
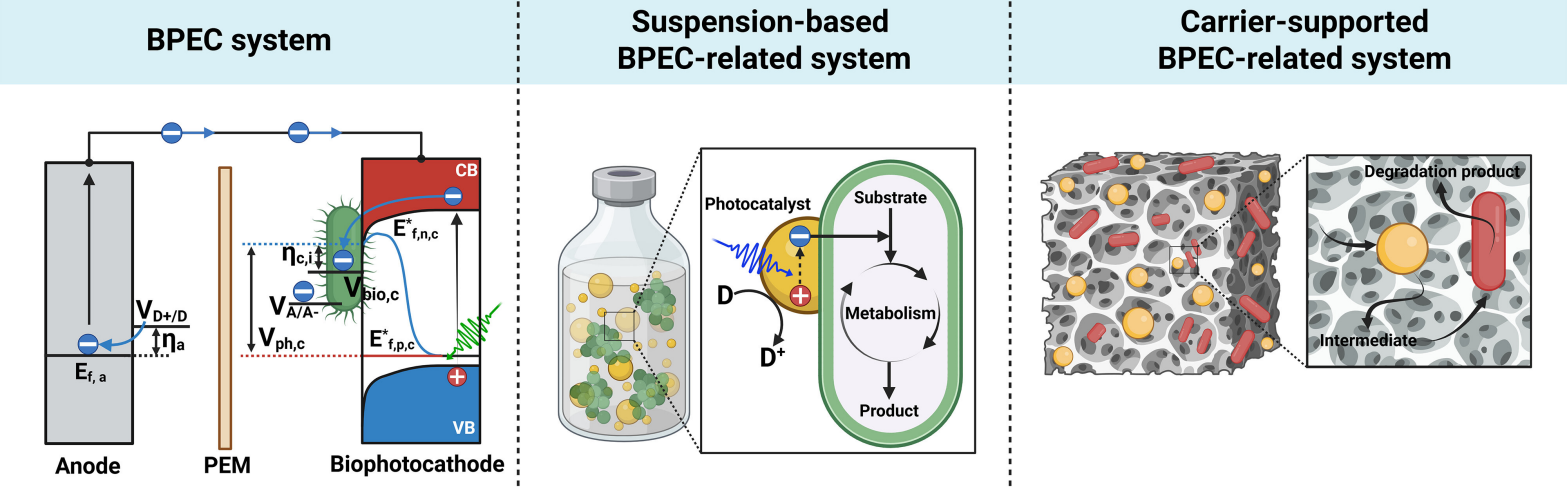
Schematic illustration of BPEC systems and suspension‐based and carrier‐supported BPEC‐related systems. Created in Biorender.

In contrast, BPEC systems consist of electrodes that can be addressed electrically. Photoactive materials, biocatalysts, or both can be combined with these electrodes. This electrical addressability allows the electrode potential to be controlled, which assists the reaction when required. Compared with BPEC‐related systems, BPEC systems offer quantifiable reaction rates, simplified product separation, and scalability. Furthermore, their flexibility in supporting different electrode architectures enables the coupling and driving of many reactions simultaneously. BPEC systems can be divided into two categories: separated‐electrode architectures, which allow for independent optimization of anodic and cathodic reactions [46, 47], and biophotoelectrode architectures, in which photoactive materials are directly interfaced with biocatalysts to minimize energy loss and enhance electron transfer (ET) efficiency [48, 49]. These features position the BPEC system as the most promising platform for the practical implementation of sustainable energy and environmental technologies.

However, existing reviews of BPEC systems lack detailed schematic band diagrams of the electrodes, which restrict the mechanistic understanding of their working principles [50]. Other reviews emphasize ET mechanisms [38] or material selection [30], leaving a knowledge gap regarding the fundamental aspect of BPEC electrode architecture. This review addresses this issue by providing a systematic and critical evaluation of electrode architectures in BPEC systems, beginning with an explanation of the working principles of photoelectrodes and bioelectrodes, including their band diagrams and various configurations, from single‐to‐dual electrodes. Next, electrode architecture in BPEC systems is classified as either a separated electrode or a biophotoelectrode, and the band diagrams of both architectures are explained, along with possible configurations. Representative applications, key performance metrics, and remaining challenges are then discussed for each architecture. The goal is to provide a conceptual framework and practical guidance to advance next‐generation BPEC systems.

## 2 Photoelectrode Working Principle and Challenges

Photoelectrochemistry is the study of light‐assisted chemical reactions at the interface between semiconductor electrodes and electrolytes [51]. The main components of PEC systems include light‐absorbing photoelectrodes, an electrolyte, and a separation membrane. The photoelectrode is usually prepared by growing a photoactive material on a conductive electrode. PEC has been used for many energy and environmental applications, including HER [52], advanced oxidation pollutant removal [53], and CO_2_RR [54]. This section discusses the working principle of the photoelectrode in enough detail to understand how a photoelectrode works. For a more detailed review of the PEC field, other excellent review articles are recommended [19, 55, 56].

Consider a PEC system consisting of an n‐type photoanode immersed in an anolyte containing an electron donor compound and a conductive cathode immersed in a catholyte containing an electron acceptor compound (Figure 2a). Both chambers are separated by a proton exchange membrane (PEM). In this example, the redox potential of the donor species (*V*_D+/D_) is more positive than that of the acceptor species (*V*_A/A−_), indicating a non‐spontaneous reaction. When the electrodes are immersed, electrons flow from the photoanode to the anolyte, causing the Fermi level of the photoanode (*E*_f,a_) to align with *V*_D+/D_. This alignment results in upward band bending near the semiconductor–liquid junction [57]. A similar alignment occurs between the Fermi level of the cathode (*E*_f,c_) and *V*_A/A−_ in the cathode but without band bending [58]. Under illumination, electrons are excited from the valence band (VB) to the conduction band (CB), reducing the degree of band bending and leaving holes in the VB. In this condition, the electron and hole are described by quasi‐Fermi levels for electrons (E*_f,n,a_) and holes (E*_f,p,a_), respectively [59]. The remaining band bending at the semiconductor–liquid junction assists holes in diffusing to the photoanode surface while sweeping electrons to the back contact, which then travel to the cathode side [60]. The oxidation reaction can proceed if E*_f,p,a_ is more positive than *V*_D+/D_, and reduction occurs if E*_f,n,a_ is more negative than *V*_A/A−_. The difference between E*_f,n,a_ and E*_f,p,a_ is the photovoltage of the photoanode (*V*_ph,a_), which represents the maximum potential available to drive the redox reaction. A photoanode that requires a lower overpotential (*η*_a_) exhibits better performance for a desired reaction rate in a specific reaction because the same reaction rate can be achieved with smaller voltage loss [61]. Photocathodes typically use p‐type semiconductors that experience downward band bending (Figure 2b). Upon illumination, electron–hole pairs are generated, resulting in the photovoltage of the photocathode (*V*_ph,c_). For a desired reduction rate, a photocathode that requires a lower overpotential (*η*_c_) exhibits better performance.

**FIGURE 2:**
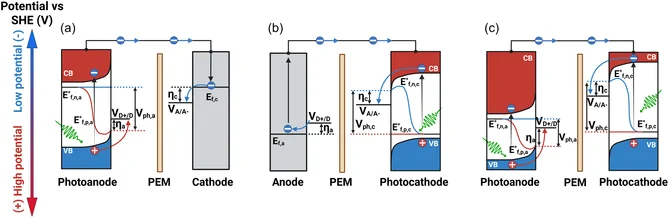
Schematic illustration of (a) photoanode, (b) photocathode, and (c) dual photoelectrode. These diagrams depict the energy alignment and charge carrier transport in an idealized manner. Created in Biorender.

In addition to a single photoelectrode, PEC systems may use a dual photoelectrode configuration consisting of an n‐type photoanode and a p‐type photocathode (Figure 2c). When illuminated, electron–hole pairs are generated in both electrodes, each creating its own quasi‐Fermi level for electrons and holes. Under the influence of band bending, minority carriers in each semiconductor migrate to the surface of the electrodes and participate in redox reactions. Meanwhile, the majority carriers flow through the external circuit to complete their path [58]. Since both electrodes are photoelectrodes, the feasibility of the oxidation is determined by the position of the hole Fermi level (*E* ^*^_f,p,a_) of the photoanode, and the reduction is determined by the electron quasi‐Fermi level (*E* ^*^_f,n,c_) of the photocathode. To facilitate electron flow from the anode to the cathode, the electron Fermi level (*E* ^*^_f,n,a_) of the anode must be more negative than the dark Fermi level of the cathode. Thus, selecting materials with appropriate Fermi‐level alignment is important when designing a dual photoelectrode configuration.

Despite the advancements in PEC research, several challenges remain and are being addressed to improve performance. For example, photoelectrode modifications via doping, pore creation, and plasmonic effects are often found in the research literature as ways to increase charge separation and reaction efficiency as well as light absorption capability [62, 63]. To boost selectivity, modification via single‐atom catalysis shows promise but has several drawbacks, such as high cost, the requirement of rare metals, and instability originating from the high surface energy of single atoms [64]. Another critical issue with photoelectrodes is photostability. Many photoelectrodes degrade under prolonged irradiation due to a lack of intrinsic self‐regeneration mechanisms. This results in rapid performance decay and the need for frequent electrode replacement [65]. Therefore, it is difficult to address energy and environmental challenges with PEC technology alone because PEC systems require sophisticated photoelectrodes that combine strong light absorption, efficient charge separation, abundant active sites, excellent photostability, and high selectivity at a low cost.

## 3 Bioelectrode Working Principle and Challenges

Bioelectrochemistry is the study of the interaction between biocatalysts and electrochemical processes [66]. In BEC, biocatalysts facilitate chemical reactions [28]. Devices in this field include microbial fuel cells (MFCs) [23], enzymatic fuel cells (EFCs) [67], microbial electrosynthesis (MES) [68], and enzymatic electro synthesis (EES) [69]. In MFCs and EFCs, biocatalyst activities convert the chemical energy of organic compounds into electricity [25]. In MES and EES, external electricity stimulates the metabolic activity of the biocatalyst and drives the production of the target chemical [70]. In all these systems, at least one electrode contains biological entities, known as a bioelectrode. The working principle of the bioelectrode is the main focus of this section, where the reader will understand how the bioelectrode works. For a more detailed review of the BEC field, other excellent review articles are recommended [71].

An MFC consisting of a bioanode and a cathode is considered, as shown in Figure 3a. For MFCs in general, the donor redox potential (*V*_D+/D_) is usually more negative than the acceptor redox potential (*V*_A/A−_). For example, glucose is a donor with an effective redox potential of −0.43 V, whereas oxygen (O_2_/H_2_O) is an acceptor with a redox potential of 0.82 V (vs. SHE (standard hydrogen electrode), pH 7). At the bioanode, microorganisms oxidize the electron donor in the electrolyte and then transfer the extracted electrons through intracellular redox chains to terminal redox‐active proteins on the cell walls of the microbes (*V*_bio,a_). Due to the close electrical contact between these redox‐active proteins and the conductive anode, electrons are transferred to the anode, setting the anode Fermi level (*E*_f,a_) slightly more positive than *V*_bio,a_ under current flow [72]. The difference between *E*_f,a_ and *V*_bio,a_ is defined as the interfacial anodic overpotential *η*_a,I_ (assuming that electrode–biocatalyst ET is the rate‐limiting step). This overpotential *η*_a,i_ can also be thought of as the voltage loss required to make current flow at the desired current density. An ideal anode material can easily accept electrons from the biocatalyst, allowing a given current density to be sustained with a small *η*_a,i_. The cathode Fermi level (*E*_f,c_) is constrained by the need to accept the electron flux from the anode while driving the reduction of the electron acceptor in the catholyte_._ A kinetic barrier in the cathode manifests as a cathodic overpotential (*η*_c_), while the operating cell voltage (*V*_OP_) is the difference between the anode and cathode potentials.

**FIGURE 3:**
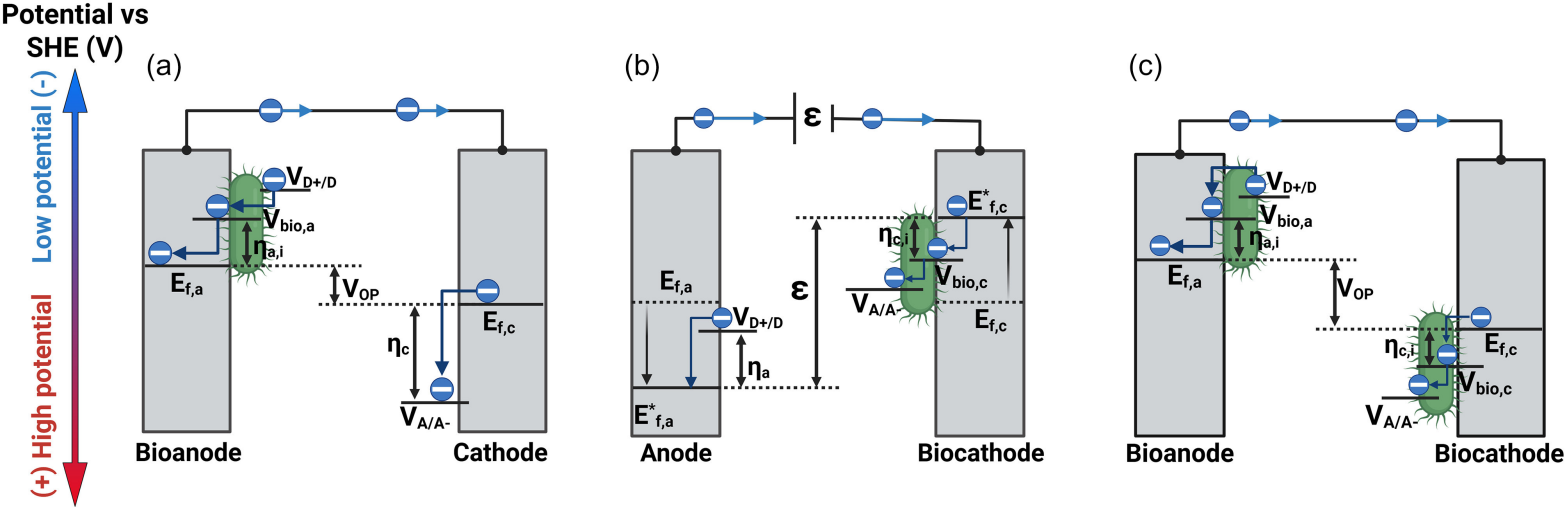
Schematic illustration of (a) a bioanode, (b) a biocathode, and (c) a dual bioelectrode. These diagrams illustrate the energy alignment and charge carrier transport in an idealized manner. Created in Biorender.

Figure 3b shows a MES cell with identical electrodes and the corresponding energy diagram to explain the biocathode mechanism. The goal in this configuration is to drive reduction at the cathode and oxidation at the anode. However, because the redox potential of the electron acceptor (*V*_A/A−_) is more negative than that of the donor (*V*_D+/D_), the reaction is non‐spontaneous. When an external bias of *ε* is applied, the cathode Fermi level (*E*_f,c_) shifts to a more negative value (E*_f,c_), the anode potential (*E*_f,a_) becomes more positive (E*_f,a_), and the potential difference between the two electrodes increases. On the anode side, holes at *E*_f,a_ can accept electrons from the donor species at *V*_D+/D_, while electrons in the cathode are transferred to the biocatalyst through redox‐active proteins or mediators at *V*_bio,c_ and finally into the electron acceptor at *V*_A/A−_. In the ideal thermodynamic limit, the reaction can proceed if the applied bias equals the difference between *V*_A/A−_ and *V*_D+/D_. In practice, however, kinetic barriers at the anode and biocathode introduce anodic and cathodic overpotentials (*η*_a_ and *η*_c,i_) that are necessary for reasonable reaction rates.

The working principle of a dual bioelectrode is essentially the same as that of a single bioanode, but it has an additional biocatalyst on the cathode side (Figure 3c). Electrons extracted on the anode side are accepted by the biocatalyst on the cathode side rather than by the acceptor species. Therefore, optimizing ET at the electrode–biocatalyst interface is the most crucial factor in dual bioelectrodes as it lowers the anodic (*η*_a,i_) and cathodic (*η*_c,i_) overpotentials.

Despite their promise, BEC systems face several key challenges that limit their performance in energy and environmental applications. One primary issue is the inherently slow reaction rate of biological catalysts. This causes long start‐up time and low current generation for MFCs as well as EFCs and also low production rates in MESs and EESs. This limitation arises because living microorganisms prioritize survival strategies over chemical conversion rates [30]. Another critical challenge is the ET at the biocatalyst–electrode interface, which can be classified as direct electron transfer (DET) and mediated electron transfer (MET) [73].

In an enzyme‐based biocatalyst, DET can occur when the redox‐active cofactor is properly oriented and close enough to the electrode surface [74]. If the cofactor is buried within the protein or not effectively connected to the electrode, a redox mediator is generally required for the ET pathway. In whole‐cell biocatalysts, the ET mechanism depends on the electroactivity of the microorganism. Exoelectrogenic bacteria, such as *Geobacter sulfurreducens*, can conduct DET through an outer membrane‐bound redox‐active protein such as a cytochrome [75] or an outer membrane extension such as a nanowire [76]. Other well‐known exoelectrogenic bacteria, such as *Shewanella oneidensis*, conduct MET via secreted flavins [77]. In contrast, non‐electroactive microorganisms, such as *Escherichia coli*, typically require an externally supplied redox shuttle such as methyl viologen.

Although DET can minimize mediator‐associated potential losses and side reactions, it is restricted by the limited number of enzymes or cells that are properly wired to the electrode surface. On the other hand, MET can extend ET beyond the electrode–biocatalyst interface to a thick biofilm. However, mediator diffusion, toxicity, and competing reactions may reduce the overall efficiency.

When choosing a biocatalyst, the trade‐offs between enzyme‐ and whole‐cell‐based biocatalysts must be considered. Although DET in enzyme‐based biocatalyst enables extremely selective and efficient ET, they require tedious and expensive purification processes that are prone to degradation [78]. On the other hand, whole‐cell biocatalysts are easier to handle, regenerative, and more robust but are limited to ET processes mainly within the biofilm [79].

## 4 Biophotoelectrochemical Systems

Although promising, PEC and BEC alone have drawbacks that limit their performance in energy and environmental applications. Interestingly, the advantages of PEC cover the disadvantages of BEC and vice versa. For example, it is difficult to drive N_2_ fixation with PEC because the N≡N bond (~945 kJ mol^−1^) [80] is highly inert, and many inorganic photocatalysts exhibit low activity and poor selectivity [81]. By contrast, nitrogenase‐bearing microorganisms can convert N_2_ to NH_3_ under ambient conditions with high selectivity [82]. Conversely, the rate‐limiting step in BEC devices, such as MFCs, is associated with EET at the microbe–electrode interface [83]. Integrating a photocatalyst to supply photogenerated holes can boost the EET rate and increase the overall current density [84]. BPEC systems combine the advantages of PEC and BEC by connecting a photoelectrode with a bioelectrode or a biophotoelectrode. Previous studies have demonstrated this concept [85, 86, 87, 88].

### 4.1 Separated Electrode Architecture

In this architecture, both the photoelectrode and the bioelectrode are connected, resulting in two possible configurations: photoanode | biocathode and bioanode | photocathode. Compared to PEC, this system can lower the amount of applied voltage needed to drive the reaction due to the synergistic effect of light energy and biological activity while maintaining the high selectivity and regeneration ability of the biocatalyst [89].

#### 4.1.1 Photoanode | Biocathode Configuration

The working mechanism of the photoanode | biocathode configuration is shown in Figure 4a. Under operating conditions, photogenerated electrons from the photoanode travel through the external circuit to the biocathode. There, they are consumed by the biocatalyst (represented by the redox potential *V*_bio,c_) to reduce the acceptor species (represented by the redox potential *V*_A/A−_). At the anode, photogenerated holes migrate to the anolyte and oxidize the donor species (*V*_D+/D_). To achieve bias‐free operation at the desired reaction rate, E*_f,n,a_ must be more negative than *V*_bio,c_, such that, under operating conditions, the resulting difference corresponds to the interfacial cathodic overpotential *η*_c,I_ required to sustain the reduction reaction (assuming that the electrode–biocatalyst interfacial ET is the rate‐limiting step). Similarly, E*_f,p,a_ must be sufficiently more positive than *V*_D+/D_ such that, under operating conditions, the resulting difference corresponds to the anodic overpotential *η*_a_ required to sustain the oxidation reaction. Therefore, factors, including the donor and acceptor species, band position, bandgap, Fermi level, light source, and the biocathode–biocatalyst interface, affect the overall performance of this configuration. The complementary effect of this configuration can be viewed in two ways depending on the reaction of interest. If the reaction of interest is a reduction, the photoanode acts as an additional energy source that enables bias‐free operation. Conversely, if the reaction of interest is an oxidation, the biocathode acts as an electron sink. This improves charge separation at the anode and enhances the hole‐driven oxidation reaction. Based on the distinction mentioned in Section 3, the examples in this section are separated according to whether the biocathode employs a purified enzyme or a whole‐cell biocatalyst.

**FIGURE 4:**
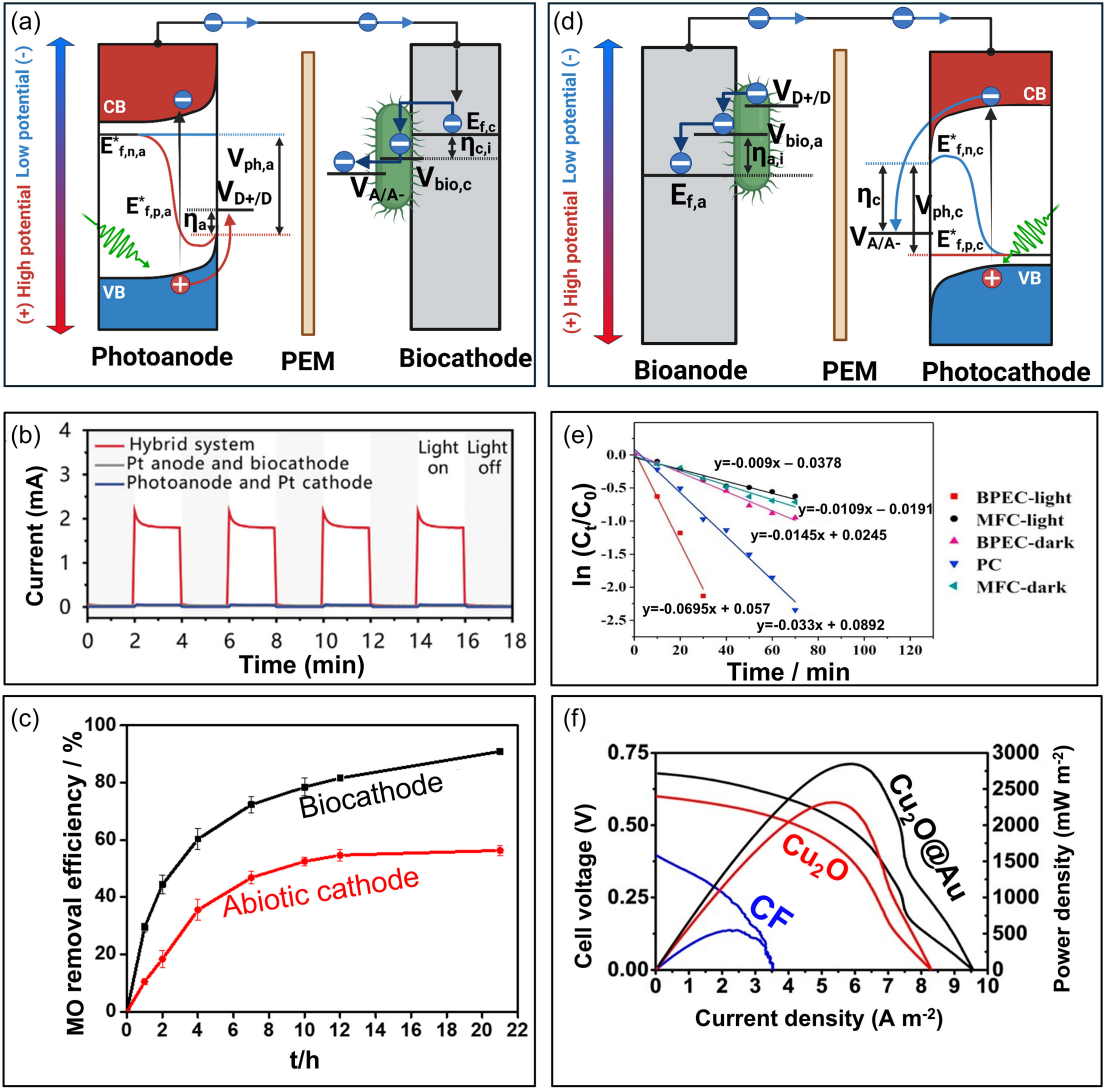
(a) Illustration of the energy bands and working principle of the photoanode | biocathode configuration. Created with Biorender.com. (b) The time profile of photocurrents for different electrode combinations in CO_2_ methanogenesis shows that a noticeable current is produced only when the biocathode is paired with a photoanode, indicating that the photoanode drives the biocathode reaction. Reproduced with permission from ref. [90] Copyright 2020, Elsevier. (c) Time dependence of removal efficiency: Coupling a biocathode with an existing photoanode improves methyl orange (MO) photodegradation efficiency by 35% compared to an abiotic cathode. Reproduced with permission from ref. [91] Copyright 2020, Elsevier. (d) Schematic illustration of the bioanode | photocathode working principle. Created using Biorender.com. (e) VOC degradation plot: The combination of a bioanode and a photocathode under illumination (BPEC‐light) exhibits the highest rate constant for VOC photooxidation. Reproduced from ref. [92] Copyright 2018, Elsevier. (f) Polarization and power curves: A visible‐light‐ active photocathode (Cu_2_O@Au) significantly increases the power output of MFC (CF) cathode. Reproduced with permission from ref. [93] Copyright 2019, Elsevier.

Purified, enzyme‐based biocathodes coupled with photoanodes can drive selective reduction reactions while decreasing the required external bias. For example, highly selective electrocatalytic CO_2_‐to‐formate conversion can be achieved using formate dehydrogenase (FDH), an enzyme that extracts electrons from a negatively charged electrode. Sokol et al. [94] used a biological light harvester (PS‐II) as an electron‐donating photoanode for an FDH‐based biocathode for CO_2_‐to‐formate conversion. Photocurrent density measurements at a 0.3 V bias demonstrate that the photoanode enhanced the current density response by seven times compared to a plain conductive anode. This highlights that the photoanode significantly boosts electron production to drive the reaction at the biocathode. Upon light irradiation and a bias of 0.3 V, PS‐II oxidized water molecules and donated the extracted electrons to the biocathode. There, CO_2_ was converted to formate with 70.6% Faradaic efficiency. The system was further modified by replacing the anode with a dye‐sensitized mesoporous TiO_2_ photoanode and the electron donor with 4‐methylbenzyl alcohol instead of water [95]. As a result, unbiased CO_2_‐to‐formate conversion was achieved with a Faradaic efficiency of 74%.

Hydrogen (H_2_) is considered a future clean energy source due to its high energy density and clean post‐combustion products [96]. Hydrogenase (H_2_ase) is an attractive enzyme‐based biocatalyst for H_2_ production because it is highly selective and efficient under mild conditions when an appropriate electron donor is available [97]. For example, PS‐II was integrated as a light harvester with a dye‐sensitized electrode to create a photoanode wired to an H_2_ase biocathode. This design achieved a significantly higher Faradaic efficiency of 76%, which was attributed to the superior water oxidation efficiency of PS‐II compared to that of inorganic photoanodes [98].

The nitrogenase enzyme can operate at ambient temperatures and pressures to convert inert N_2_ to NH_3_, provided that electrons are delivered to its active site in a controlled manner. Meirovich et al. [99] developed a light‐driven BEC cell with a CdS/NiO photoanode as an electron donor for a nitrogenase‐based biocathode. The photoanode oxidized glucose, and the extracted electrons moved to the cathode chamber, where the electron shuttle (SPr)_2_V—structurally known as 1,1′‐bis(3‐sulfonatopropyl)‐4,4′‐‍bipyridinium) accepted and delivered them to the nitrogenase enzyme. As a result, 100 µM ammonia solution was produced after 2 h of operation under bias‐free conditions. These studies highlight the role of the photoanode in aiding the thermodynamically uphill reduction by minimizing the required bias or enabling bias‐free operation.

The photocatalytic degradation of highly toxic and stable pollutants such as 4‐nitrophenol (4‐NP) is typically UV‐driven and kinetically limited [100]. To enhance visible‐light performance, one study coupled a strong, visible‐light‐responsive Ag_3_PO_4_/BiVO_4_ photoanode with a horseradish peroxidase (HRP) biocathode [101]. Under visible‐light irradiation, the photoanode generated reactive oxygen species (•OH and O_2_•^−^), and photogenerated electrons were transferred to the biocathode, which reduced O_2_ into H_2_O_2_. This activated HRP and accelerated 4‐NP degradation. This synergistic process achieved 97.1% removal of 10 mg L^−1^ 4‐NP within 60 min. In addition to eliminating oxidation intermediates, another study valorized CO_2_, a common by‐product of organic oxidation, by coupling a TiO_2_@CdS photoanode for bisphenol‐A (BPA) degradation with an FDH‐based biocathode for CO_2_‐to‐formate conversion [102]. This system achieved 78% BPA degradation and 50% CO_2_ recovery after 3.5 h. These studies highlight the role of the biocathode in assisting with photooxidation reactions, particularly in environmental remediation and energy recovery applications.

Whole‐cell‐based biocathodes have also been integrated with photoanodes to drive multistep, complex chemical reactions while maintaining high selectivity. For example, unlike the two‐electron CO_2_‐to‐formate conversion, CO_2_‐to‐CH_4_ conversion is an 8‐ET reaction that requires more energy and is typically driven by a whole‐cell biocatalyst (e.g. methanogens) that uses H_2_ or electrode‐supplied electrons [103]. One study used a TiO_2_/CdS heterostructure photoanode to supply electrons to methanogens immobilized on a chitosan‐modified carbon cloth biocathode [90]. The photoanode supplied electrons to the methanogens since current was only observed when the photoanode and biocathode were used together (hybrid system), as shown in Figure 4b. Using this configuration, a solar‐to‐fuel (STF) conversion efficiency of up to 1.28% was achieved, with a Faradaic efficiency of 94.4%.

Replacing conventional conductive cathodes with whole‐cell biocathodes can enhance the efficiency and functionality of photocatalytic oxidation systems by taking advantage of the cells’ ability to decompose small organic matter intermediates derived from photooxidation. For example, dyes such as methyl orange often transform into small organic intermediates during photocatalytic degradation, which hinder further oxidation and limit overall removal efficiency. One study addressed this issue by coupling a TiO_2_/g‐C_3_N_4_ photoanode with a denitrifying biocathode [91]. Under light irradiation and an applied bias of −0.5 V (vs. Ag/AgCl), the hybrid system achieved 35% higher methyl orange degradation efficiency than an abiotic cathode (Figure 4c). This improvement was attributed to the rapid biodegradation of oxidation intermediates by denitrifying bacteria, which sustained the photocatalytic reaction.

Another strategy employed genetically engineered *E. coli* that express H_2_ase, thereby improving stability. The modified cells were immobilized on a biocathode paired with a TiO_2_ photoanode. Under 365 nm irradiation, photogenerated holes at the TiO_2_ photoanode oxidized water, while electrons were transferred through the external circuit to the *E. coli* via a methyl viologen electron shuttle. This enabled microbial H_2_ production at a rate of 1.88 × 10^−7^ mol h^−1^ and a Faradaic efficiency of 11.9% [104]. The Faradaic efficiency in this study was significantly lower than in a study that used an enzyme‐based biocatalyst (H_2_ase) [98]. This might be attributed to the fact that H_2_ase was embedded inside *E. coli* and used MET mode, which decreased ET efficiency. Additionally, a pristine TiO_2_ anode was used with *E. coli*, whereas the other study used a PS‐II‐based anode, which significantly boosted the water oxidation reaction and enhanced the overall reaction. A summary of the photoanode | biocathode configuration is shown in Table 1.

**TABLE 1:** Summary of the photoanode | biocathode configuration (DA, 1,1′‐diaminoethyl‐4,4′‐bipyridinum; dpp, phosphonated diketopyrrolopyrrole dye; FE, Faradaic efficiency; FDH, formate dehydrogenase; FTO, fluorine tin oxide; IO, Inverse opal; NR, not reported; PANi, polyaniline; P_os_, [poly(1‐vinylimidazole‐*co*‐allylamine)‐[Os(bipy)_2_Cl]Cl redox polymer; PSII, photosystem II; rGO, reduced graphene oxide; STF, solar‐to‐fuel efficiency; STH, solar‐to‐hydrogen efficiency). The reported electrode size is either the physical size or the projected surface area.

Biocatalyst/ET mode	Application	Electrode (size) and working volume (WV)	Light source	Performance	References
Enzyme/DET	CO_2_ to formate	Photoanode: IO‐TiO_2_|dpp|P_O__s_‐PSII (0.25 cm^2^), Biocathode: IO‐TiO_2_|FDH(0.25 cm^2^), WV: NR	Solar simulator (100 mW cm^−2^, >420 nm)	Bias: 0.3 V, FE: 70.6%	[94]
Enzyme/DET	CO_2_ to formate	Photoanode: FTO|mTiO_2_|dpp (0.25 cm^2^), Biocathode: FTO|mITO|FDH|(0.19 cm^2^), WV: NR	Solar simulator (100 mW cm^−2^, >420 nm)	Unbiased, FE: 74%	[95]
Enzyme/DET	H_2_ generation	Photoanode: FTO|IO‐TiO_2_|dpp|P_os_‐PSII (0.25 cm^2^), Biocathode: FTO|IO‐TiO_2_|H_2_ase (0.25 cm^2^), WV: 12 mL	150 W Xe lamp (100 mW cm^−2^, >420 nm)	Unbiased, STH: 0.14%, FE: 76%	[98]
Enzyme/MET	N_2_ to NH_3_	Photoanode: CdS/NiO (NR), Biocathode: nitrogenase (NR), WV: 4.5 mL	5 W white LED	Unbiased, FE: 12%	[99]
Enzyme/unspecified	4‐Nitrophenol degradation	Photoanode: Ag_3_PO_4_/BiVO_4_ (2 × 2 cm), Biocathode: horseradish peroxidase (HRP) (NR), WV: NR	300 W Xe lamp (200 mW cm^−2^, >420 nm)	Unbiased, Pollutant concentration: 10 mg L^−1^, Rate constant: 5.89 × 10^−2^ min^−1^	[101]
Enzyme/MET	BPA removal, electricity generation, and CO_2_ to formate conversion	Photoanode: TiO_2_@CdS (7 cm^2^), Biocathode: FDH/DA/PANi/carbon cloth (5 cm^2^), WV: 16 mL	300 W Xe lamp (5.7 mW cm^−2^, >420 nm)	Unbiased, Pollutant concentration: 70 mg L^−1^, Rate constant: 1.514 h^−1^, CO_2_ recovery efficiency: 47.4%, Power density: 94.1 mW m^−2^	[102]
Whole cell/DET	CO_2_ to CH_4_	Photoanode: TiO_2_/CdS (2.5 × 1.2 cm), Biocathode: mixed methanogens on carbon cloth (4 × 5 cm^2^), WV: 600 mL	Solar simulator (75 mW cm^−2^, AM1.5G)	Unbiased, STF: 1.28%, FE: 94.4%	[90]
Whole cell/unspecified	Methyl orange removal	Photoanode: TiO_2_/g‐C_3_N_4_/rGO (15 cm^2^), Biocathode: denitrifying bacteria (9 cm^2^), WV: 150 mL	300 W Xe lamp	Bias: −0.5 V versus Ag/AgCl, Pollutant concentration: 100 mg L^−1^, Rate constant: 0.11 h^−1^	[91]
Whole cell/MET	H_2_ generation	Photoanode: TiO_2_ (1.5 × 1.5 cm), Biocathode: H_2_ase‐bearing *E. coli* (1–10 cm^2^), WV: 150 mL	LED light (279 mW cm^−2^, 365 nm)	Unbiased, HER rate: 0.188 µmol h^−1^, FE: 11.9%	[104]

#### 4.1.2 Bioanode | Photocathode Configuration

The working mechanism of this configuration is shown in Figure 4d. Since the redox potential of the donor species (*V*_D+/D_) is more negative than that of the acceptor species (*V*_A/A−_), the reaction is spontaneous. The biocatalyst on the bioanode extracts electrons from the donor species and transports them to the photocathode via an external circuit. There, the electrons neutralize the photogenerated holes inside the photocathode. Meanwhile, photogenerated electrons in the photocathode migrate to the surface of the semiconductor, where they reduce the acceptor species. For spontaneous reactions, such as the one depicted here, current can still flow in the dark if *E*_f,a_ is sufficiently negative to provide the required reduction overpotential. If the reaction of interest occurs at the photocathode, the bioanode mainly enhances charge separation by neutralizing photogenerated holes. Conversely, when the reaction of interest is a biological oxidation, the photocathode lowers the required overpotential to run the oxidation at the same rate.

Replacing a conventional conductive anode with a bioanode in PEC devices could improve the photocatalytic reduction reaction and potentially provide additional functionality such as power generation. For example, a microbial‐colonized bioanode was used to enhance charge separation in a rutile TiO_2_ photocathode, thereby augmenting the efficiency of Cr(VI) removal [46]. The microorganisms spontaneously oxidized acetate, causing electrons (from acetate oxidation) to travel to the photocathode via an external wire. There, the electrons recombined with photogenerated holes, thereby improving charge separation in TiO_2_. Consequently, the isolated photogenerated electrons migrated to the photocathode surface and improved the reduction efficiency of Cr(VI) to Cr(III) by about fivefold compared to an abiotic anode. A similar mechanism was proposed by Ahmadpour et al. [105], who coupled a microbial‐colonized bioanode with an AgBr/ZnO heterostructure photocathode to degrade azo dyes. Improved electron–hole separation due to microbial activity increased the availability of electrons to generate reactive organic species that degraded the azo dye. This device achieved 61% dye decolorization, 13% higher than using an abiotic anode. Additionally, this design generated 53.87 mW m^−2^ of power density, demonstrating the potential to treat waste and generate clean energy simultaneously within a single system.

Volatile organic compounds (VOCs) are a major air pollutant due to their toxic effects on the environment and human health [106]. One study used a *S. oneidensis* bioanode to assist in the rapid cathodic degradation of VOCs using a TiO_2_ photocathode and promote electricity production simultaneously. Electrons extracted from organic compounds were consumed by photogenerated holes inside TiO_2_, thus improving charge separation [92]. Therefore, the excited electrons could react with oxygen from the air to form O_2_^•^^−^ radical anions, which degraded ethyl acetate. Consequently, the degradation kinetic constant of this design (BPEC‐light) was twofold higher than that of the photocatalyst only (PC) and nearly eightfold higher than that of the biocatalyst only (MFC‐light), as shown in Figure 4e.

Bias‐free photoelectrocatalytic H_2_ production and CO_2_ reduction typically use dual photoelectrode systems that require complex material design and engineering [107]. One way to overcome these challenges is to use a bioanode that can supply electrons to the photocathode. One study used a microbial‐colonized bioanode coupled with a MoS_3_‐deposited B‐doped Si nanowire photocathode for H_2_ evolution. This bioanode–photocathode couple generated a voltage that was 68 times higher than that of a plain anode‐photocathode couple, which highlights the role of the bioanode in increasing electron availability. Consequently, a H_2_ production rate of 7.5 µmol h^−1^ cm^−2^ with a maximum power density of 71 mW m^−2^ was achieved [108].

Recently, a study demonstrated the possibility of using output power from microbial oxidation to enable bias‐free CO_2_‐to‐syngas conversion using a power management circuit (PMC). In this work, the researchers coupled a bioanode rich in microbes with a photocathode made of Si nanowire embedded with a single‐atom Ni catalyst (Si NW/Ni SA) [109]. It was demonstrated that the selectivity of CO_2_ to CO or H_2_ could be easily adjusted by changing the voltage applied to the photocathode from −0.1 to −0.7 V versus RHE through the PMC. Using this strategy, a CO/H_2_ ratio of 0.6 and a total Faradaic efficiency of 96.6% were obtained. Altogether, these examples show the role of the bioanode in enhancing photocatalytic reduction reactions.

In MFCs, the oxygen reduction reaction at the cathode often limits performance due to sluggish four‐ET kinetics. Replacing conventional conductive cathodes with photocathodes can provide additional photovoltage to enhance power output. For instance, coupling a rutile photocathode with a microbial‐rich bioanode significantly increased power density, achieving approximately twice the output of an MFC with a graphite cathode [110]. Further improvements were realized with a Bi_2_O_3_/TiO_2_ photocathode responsive to visible light. In this system, photoexcited electrons were injected from the Bi_2_O_3_ to the TiO_2_ CB, enhancing charge separation. Under visible‐light irradiation, this system delivered a power density nearly three times higher than that of a conventional activated carbon cathode [111].

Enhancing MFC performance further has been achieved by exploiting the plasmonic effects of metal nanoparticles to broaden light absorption. In one study, a three‐dimensional Cu_2_O@Au nanowire photocathode was connected to an N‐doped carbon bioanode colonized by *S. oneidensis* [93]. Due to plasmonic excitation of the Au, which injected hot electrons into the Cu_2_O and improved charge separation, the system produced a power output more than four times higher than that of a carbon felt cathode (Figure 4f). Beyond power generation, photocathodes can enhance the biodegradation of pollutants. For example, a Bi_2_O_3_/CuO photocathode promoted chlorotetracycline (CTC) degradation by generating hydroxyl and carbonate radicals. Working synergistically with a microbial‐colonized bioanode, it achieved 85.4% CTC removal with a rate constant of 0.0066 min^−1^ [112]. Replacing this photocathode with a CuO/CdS quantum dot‐based material doubled the degradation rate constant to 0.01242 min^−1^ while maintaining a similar overall removal efficiency after 12 h of operation [113]. A summary of the bioanode | photocathode configuration is shown in Table 2.

**TABLE 2:** Summary of the bioanode | photocathode configurations (AM1.5G, air mass 1.5 global; FE, Faradaic efficiency; NR, Not reported; NW, nanowire; SA, single atom). The reported electrode size is either the physical size or the projected surface area.

Biocatalyst/ET mode	Application	Electrode (size) and working volume (WV)	Light source	Performance	References
Whole cell/unspecified	Cr removal and energy recovery	Bioanode: microbial‐rich graphite (5 × 7 cm), Photocathode: rutile (TiO_2_) (5 × 7 cm), WV: 510 mL	Solar simulator (12.8 mW cm^−2^, IR‐cutoff)	Pollutant concentration: 26 mg L^−1^, Removal efficiency: 97%	[46]
Whole cell/unspecified	Reactive black 5 degradation and energy recovery	Bioanode: microbial‐colonized graphite plate (3 × 3 cm), Photocathode: AgBr/ZnO (3 × 3 cm), WV: 440 mL	20 W LED bulb (>420 nm)	Pollutant concentration: 10 mg L^−1^, Degradation efficiency: 61% after 72 h, Max. power density: 53.87 mW m^−2^	[105]
Whole cell/unspecified	VOC removal and energy recovery	Bioanode: microbial‐rich graphite rod (∅ 0.5 × 12 cm), Photocathode: TiO_2_ (3 × 10 cm), WV: 60 mL	9 W UV light (3.5 mW cm^−2^, 365 nm)	Pollutant concentration: 125 ppm, Rate constant: 0.085 min^−1^, Max. power density: 92.8 mW cm^−2^	[92]
Whole cell/ unspecified	H_2_ production and energy recovery	Bioanode: microbial‐colonized carbon felt (16 cm^2^), Photocathode: MoS_3_‐B Si NW (6 cm^2^), WV: 215 mL	350 W Xe lamp (12 mW cm^−2^)	Unbiased, HER rate: 7.5 µmol h^−1^ cm^−2^, Max. power density: 71 mW m^−2^	[108]
Whole cell/unspecified	CO_2_ to syngas	Bioanode: microbial‐rich carbon fiber brush (∅ 2.5 × 2.5 cm), Photocathode: Si NW/Ni SA (0.15–0.4 cm^2^), WV: 180 mL	300 W Xe lamp (100 mW cm^−2^, AM1.5G)	Unbiased, FE CO: 81%, Ratio of CO to H_2_: 0.1–0.68	[109]
Whole cell/unspecified	Energy recovery	Bioanode: microbial‐rich graphite (5 × 6 cm), Photocathode: TiO_2_‐coated graphite (5 × 6 cm), WV: 620 mL	Solar simulator (12.8 mW cm^−2^)	Max. power density: 60.15 mW m^−2^	[110]
Whole cell/unspecified	Energy recovery	Bioanode: microbial‐rich carbon felt (NR), Photocathode: Bi‐doped TiO_2_ (NR), WV: NR	5 W LED bulb	Max. power density: 224 mW m^−2^	[111]
Whole cell/unspecified	Energy recovery	Bioanode: *S. oneidensis* on N‐doped carbon (∅ 0.9 × 0.15 cm), Photocathode: Cu_2_O@Au NW (1 × 1 cm), WV: 200 mL	500 W Xe lamp (AM1.5G)	Max. power density: 2315.7 mW m^−2^	[93]
Whole cell/unspecified	CTC degradation	Bioanode: microbial‐colonized graphite brush (NR), Photocathode: Bi_2_O_3_/CuO (NR), WV: 145 mL	300 W Xe lamp	Pollutant concentration: 20 mg L^−1^, Rate constant: 0.0066 min^−1^	[112]
Whole cell/unspecified	CTC degradation	Bioanode: microbial‐colonized graphite brush (NR), Photocathode: CuO/CdS S‐scheme quantum dots (NR), WV: 145 mL	300 W Xe lamp	Pollutant concentration: 20 mg L^−1^, Rate constant: 0.01242 min^−1^	[113]

### 4.2 Biohybrid Photoelectrode Architecture

A biohybrid photoelectrode (biophotoelectrode) consists of a photoelectrode in contact with a biocatalyst. It can be used as either an anode (biohybrid photoanode or biophotoanode) or a cathode (biohybrid photocathode or biophotocathode). In a biophotoelectrode, the photoelectrode harvests light energy while the biocatalyst carries out the reaction of interest.

#### 4.2.1 Biophotoanode Configuration

The working principle of a biophotoanode is schematically shown in Figure 5a. The biocatalyst at the interface extracts electrons from the donor species (represented by the redox potential *V*_D+/D_), which are then accepted by the photogenerated holes at the photoanode surface. Meanwhile, the photogenerated electrons travel to the cathode via the external circuit. Unlike a conventional bioanode, where the anode Fermi level is determined by the dynamics of electron injection from the biocatalyst and ET to the cathode, the biophotoanode allows these processes to be decoupled. Under illumination, interfacial electron injection is governed by the hole quasi‐Fermi level (E*_f,p,a_), while ET to the cathode is facilitated by the electron quasi‐Fermi level (E*_f,n,a_). As a result, the same oxidation rate can be achieved with a lower overpotential while maintaining a sufficient driving force for ET to the cathode. This configuration can consequently enhance electron extraction from the biocatalyst and improve the achievable power output in MFC devices, provided that recombination and interfacial losses are effectively controlled.

**FIGURE 5:**
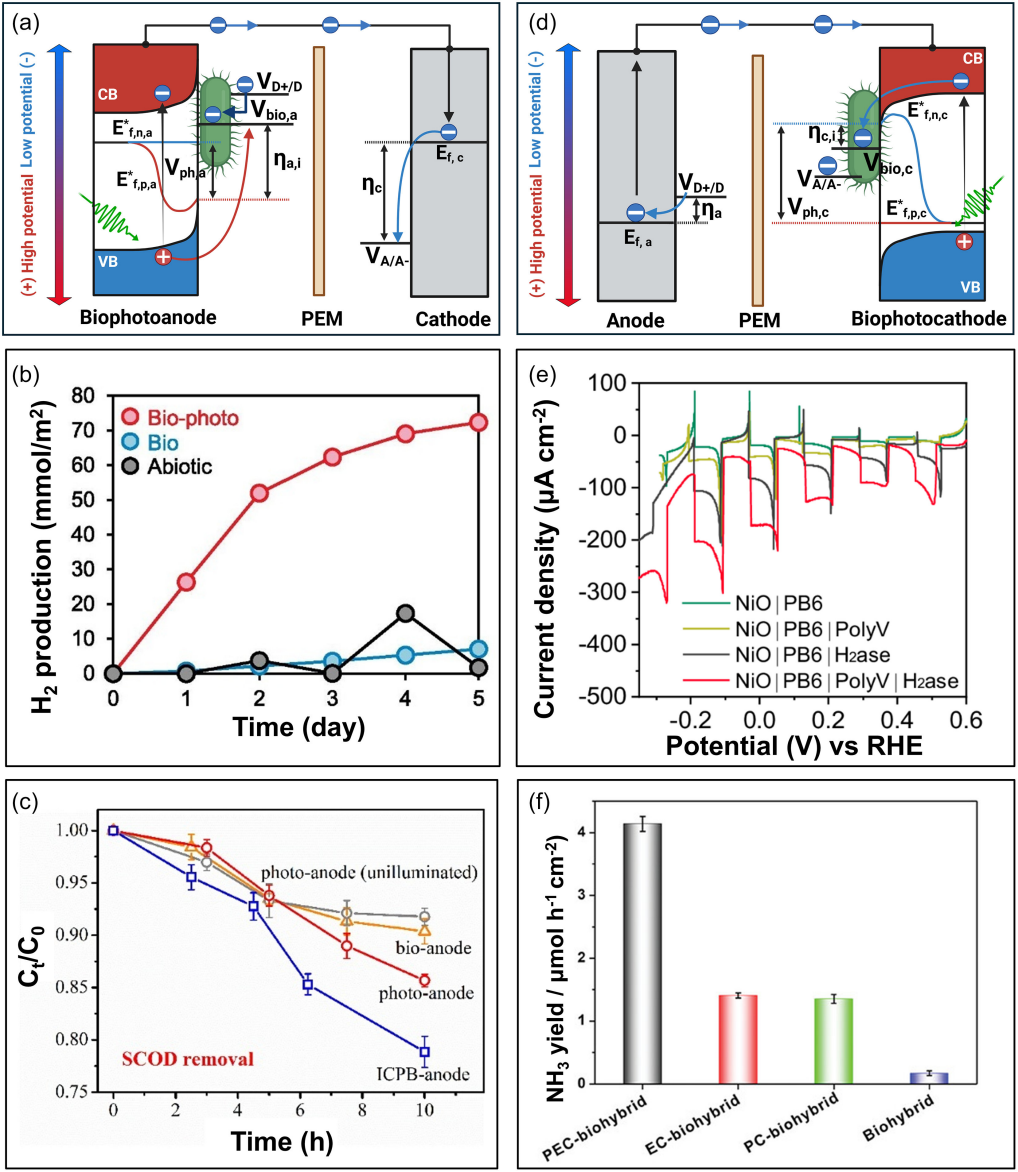
(a) Schematic illustration of the working principle of biophotoanodes. Created with Biorender. (b) Time dependence of H_2_ production: HER only occurs when the biophotoanode is paired with a cathode at −0.24 V versus RHE. This suggests that the biophotoanode plays a role in lowering the required external energy. Reproduced with permission from ref. [33] Copyright, 2025 Elsevier. (c) The soluble chemical oxygen demand profile from 4‐CP degradation using different anode materials shows that the biophotoanode has the highest removal efficiency. Reproduced with permission from ref. [114] Copyright 2017 Elsevier. (d) Schematic illustration of the working principle of biophotocathodes. Created using Biorender.com. (e) Controlled potential electrolysis using chopped light: Current density using different cathode materials suggests that the biophotocathode with a viologen‐based mediator outperforms other materials. Reproduced with permission from ref. [115] Copyright 2024, Nature. (f) NH_3_ production from N_2_ using various cell concepts: The NH_3_ yield shows the excellent performance of the biophotocathode compared to other designs. Reproduced with permission from ref. [35] Copyright 2024, Wiley.

The first biophotoanode was created as proof of concept, demonstrating that a photoanode accelerates the EET process more than a conductive anode does. This biophotoanode was composed of *S. oneidensis MR1* on hematite nanowires and, with a 0.8 V bias (vs. Ag/AgCl), exhibited a 12.5 times higher current density under illumination than in the unilluminated condition [48]. Feng et al. [116] took advantage of this concept by immobilizing microorganisms onto a Fe_2_O_3_‐covered graphite plate. As a result, startup time was reduced by about 60% and current density was more than twice that of a BEC. Another study leveraged this concept by using the boosted current produced by the biophotoanode to drive HER. The biophotoanode was created by attaching *S. oneidensis* MR‐1 to CuO‐NW/ZnO/CuO‐NP; the cathode was a Pt wire [33]. Under illumination, this design produced 70 mmol m^−2^ H_2_ in 5 days (Figure 5b), corresponding to a generation rate of 13.6 mmol m^−2^ days^−1^ with only −0.24 V versus RHE applied to the Pt cathode. This result demonstrates that the biophotoanode supplies a significant portion of the required driving force through its photovoltage, while the remaining energy is supplied by organic matter oxidation. To achieve bias‐free H_2_ generation using a biophotoanode, another work coupled a biophotoanode with a photocathode. The biophotoanode was constructed by compressing an Fe_2_O_3_ photoelectrode with a microbial‐colonized graphite brush. The photocathode was made from a Pt‐deposited black Si wafer [117]. Under one sun (100 mW cm^−2^, AM1.5) irradiation, the system achieved a maximum current density of 0.8 mA cm^−2^ and an H_2_ production rate of 1224 mmol m^−2^ day^−1^. This was about 100 times higher than the previous design due to the additional power supply from the highly light‐absorbing photocathode.

In addition to HER, the biophotoanode is employed for removing pollutants and recovering energy. For example, metal oxides were impregnated onto a nanostructured, carbon‐based substrate. CeO_2_ was used for the photoanode and Cu_2_O for the photocathode [118]. Using a 100 W lamp for irradiation, the system achieved 94% chemical oxygen demand (COD) reduction and 97% Cr(VI) removal in the anode and cathode chambers, respectively, with a maximum power density of 1107 mW m^−2^. Electrochemical impedance spectroscopy of both photoelectrodes indicates reduced charge transfer resistance and more efficient interfacial electron transport. This indicates that ET from microorganisms to the photoanode improved significantly as did the overall performance.

Beyond photocurrent enhancement, several studies have utilized the proximity of photoanodes to biocatalysts to eliminate persistent pollutants via sequential photocatalytic oxidation, followed by biodegradation [119]. Long et al. [120] used WO_3_/TiO_2_ nanotubes attached to carbon felt as a biophotoanode and a graphite cathode to degrade aniline. They achieved total organic carbon (TOC) removal efficiencies 24% and 43% higher than those of photocatalytic or microbial degradation alone, demonstrating a strong synergistic effect. In another study, N‐TiO_2_ nanopowder and anode‐respiring bacteria were immobilized on a carbon foam electrode, which served as the biophotoanode (referred as intimate coupling of photocatalysis and biodegradation‐anode or ICPB anode). A stainless‐steel mesh was used as the cathode to simultaneously degrade 4‐chlorophenol (4‐CP) and generate electricity [114]. ICPB anode successfully protected the microbes from reactive oxygen species, enabling further degradation of oxidation intermediates. ICPB anode degradation rate of 4‐CP was 1.6‐fold higher than with bioanode and ~20% higher than with photoanode. This design also achieved the highest soluble COD removal, indicating superior elimination of photooxidation intermediates from 4‐CP (Figure 5c). A summary of the biophotoanode configuration is shown in Table 3.

**TABLE 3:** Summary of biophotoanode configurations (ACF, activated carbon fiber; AM1.5G, air mass 1.5 global; ARB, anode respiring bacteria; CA, carbonic anhydrase; CNFs, carbon nanofibers; COD, chemical oxygen demand; NP, nanoparticle; NR, not reported; NT, nanotube; TOC, total organic carbon). The reported electrode size is either the physical size or the projected surface area.

Biocatalyst/ET mode	Application	Electrode (size) and working volume (WV)	Light source	Performance	References
Whole cell/unspecified	Energy recovery	Biophotoanode: *S. oneidensis* on Fe_2_O_3_ (1.0–1.5 cm^2^), Cathode: Pt wire (NR), WV: NR	40 mW cm^−2^, AM1.5G	Photocurrent: 250 µA cm^−2^	[48]
Whole cell/unspecified	Energy recovery	Biophotoanode: microbial‐enriched Fe_2_O_3_‐coated graphite (12.5 cm^2^), Cathode: graphite felt (∅4 cm), WV: 80 mL	35 W Xe lamp (10 mW)	Photocurrent: 840 µA cm^−2^	[116]
Whole cell/DET	H_2_ production	Biophotoanode: *S. oneidensis* on CuO‐NW/ZnO/CuO‐NP (NR), Cathode: Pt wire (NR), WV: 120 mL	LC8 (100 mW cm^−2^, 400 < *λ* < 600 nm)	Bias ~ −0.24 V versus RHE, H_2_ production rate: 13.6 mmol m^−2^ days^−1^	[33]
Whole cell/ unspecified	H_2_ and energy production	Biophotoanode: microbial‐colonized Fe_2_O_3_ (1 × 1 cm), Cathode: Pt‐deposited black Si wafer (NR), WV: 28 mL	300 W Xe lamp (100 mW cm^−2^, AM1.5G)	Current density: 0.8 mA cm^−2^, H_2_ production rate: 1224 mmol m^−2^ days^−1^	[117]
Whole cell/unspecified	COD, Cr (VI) removal, and energy recovery	Biophotoanode: microbial‐enriched CeO_2_‐impregnated ACF/CNFs (1 × 1 cm), Cathode: Cu_2_O‐impregnated ACF/CNFs (1 × 1 cm), WV: 400 mL	100 W visible‐light lamp	COD removal after 3 days: 94% (initial concentration: 13.8 mg L^−1^), Cr(VI) removal after 3 days: 97% (initial concentration: 50 ppm), Max power density: 1107 mW m^−2^	[118]
Whole cell/unspecified	Aniline removal	Biophotoanode: Microbial‐enriched WO_3_/TiO_2_ NT (3 × 3 cm), Cathode: graphite (NR), WV: 100 mL	300 W Xe lamp	Pollutant concentration: 100 mg L^−1^, Rate constant: 0.039 h^−1^, TOC removal efficiency: 92%	[120]
Whole cell/DET	4‐CP removal and energy recovery	Biophotoanode: ARB immobilized on N‐TiO_2_‐coated carbon foam (3 × 3 cm), Cathode: stainless steel mesh (7 × 7 cm), WV: 120 mL	150 W halogen cold lamp (380 < *λ* < 800 nm)	Pollutant concentration: 25 mg L^−1^, Rate constant: 0.074 h^−1^, Current density: 40 µA cm^−2^	[114]

#### 4.2.2 Biophotocathode Configuration

The working principle of a biophotocathode is shown schematically in Figure 5d. In this configuration, the biocatalyst is immobilized directly on the p‐type semiconductor surface. When illuminated, photogenerated electrons in the CB transfer to the biocatalyst, often via redox‐active proteins, to drive reduction. Meanwhile, electrons extracted from anodic oxidation travel through the external circuit to neutralize photogenerated holes in the VB of the photocathode. This design offers distinct advantages over the photoanode | biocathode configuration described in Section 4.1.1. In the latter case, electrons must travel from the anode to the cathode, experiencing a significant voltage drop across multiple interfaces (anode wire, external wire, and wire cathode contacts). Consequently, the energy of the electrons that arrive at the cathode is lower than when they were generated, which decreases the thermodynamic feasibility of driving high‐energy reduction reactions. In contrast, a biophotocathode generates high‐energy electrons locally near the photocathode‐biocatalyst interface, thereby minimizing energy loss. Furthermore, the internal electric field created by band bending within the biophotocathode actively drives electrons toward the biocatalyst. This offers better charge transport than the passive conduction mechanisms of dark electrodes. Together, these features maximize the energy of electrons reaching the catalytic site, significantly reducing the required external bias and bringing the system closer to bias‐free operation. Examples discussed in this section distinguish between enzyme‐based and whole‐cell‐based biocatalysts, beginning with the former.

Recently, solar‐driven water splitting for HER was demonstrated using an enzyme‐based biophotocathode. One representative system employed a dye‐sensitized (Pigment Blue 6, PB6) NiO biophotocathode with immobilized H_2_ase and coupled to a BiVO_4_ photoanode. Measurement of the photocurrent density under one sun illumination revealed that the achievable photocurrent density was 141 µA cm^−2^ for the biophotocathode (NiO | PB6 | PolyV | H_2_ase) equipped with a viologen‐based electron mediator (PolyV), whereas the photocathode (NiO | PB6) produced only a negligible current density (Figure 5e). Performance tests suggest that the biophotocathode successfully produced H_2_ with 81% Faradaic efficiency and a solar‐to‐hydrogen (STH) efficiency of 0.124% [115]. To eliminate the need for viologen‐based redox mediators and associated unwanted side reactions, Yeung et al. [121] developed direct ET photocathodes by integrating organic photovoltaic (OPV) absorbers with H_2_ase immobilized on inverse opal TiO_2_ and further stabilized by carbonic anhydrase. This architecture significantly increased the photocurrent, achieving 39 µmol cm^−2^ of H_2_ production over 10 h with an STH of 0.49%. Building on this strategy, encapsulated lead halide perovskites were introduced later as high‐efficiency light absorbers in biophotocathodes. This enabled bias‐free H_2_ production with an improved STH efficiency of 1.1% when paired with a BiVO_4_ photoanode [122].

CO_2_‐to‐chemical production is an interesting area of research for realizing sustainable carbon capture and utilization technology [123]. Different products require different reaction chemistries, which can be classified by the number of electrons involved. Reactions requiring more electrons and longer carbon chains are generally more challenging. The following examples are ordered from simplest to most difficult. FDH is a highly selective enzyme that can directly convert CO_2_ to formate (C1, 2 e^−^) [124, 125]. Recently, the Reisner group demonstrated a semi‐artificial photosynthetic system that couples CO_2_‐to‐formate conversion with O_2_ evolution using an OPV‐based biophotocathode [121]. An organic light‐absorbing layer and inverse opal TiO_2_ immobilized the FDH, and carbonic anhydrase was co‐immobilized to regulate the local microenvironment and enhance stability. This mediator‐free system produced 63 µmol cm^−2^ formate over 10 h, corresponding to a STF efficiency of 0.57%.

Whole‐cell‐based biocatalysts are advantageous for driving more complex CO_2_ reduction reactions that involve many ETs or a long carbon chain final product. The conversion of CO_2_ to CH_4_ (C1, 8 electrons) was demonstrated in a bias‐free mode using a p*‐*InP/Pt photocathode immersed in a *Methanosarcina barkeri* culture and paired with a TiO_2_ photoanode [34]. Under illumination, the photoanode oxidized water to supply protons while the photocathode generated H_2_, which was subsequently consumed by *M. barkeri* for biological methanation. The illuminated system produced approximately three times more CH_4_ than the dark control, with a Faradaic efficiency of 74%, confirming the effectiveness of solar‐driven, biological CO_2_ methanation.

More complex C2 products, such as acetate (8 e^−^) and ethanol (12 e^−^), have also been realized. One study employed a low‐cost CuBi_2_O_4_ photocathode in direct contact with *Sporomusa ovata*, operating at 0 V versus SHE under illumination [126]. After 140 h, comparable concentrations of acetate and ethanol were produced (FE ~34%–35%). Although *S. ovata* physiologically prefers acetate, the initial formation of ethanol was attributed to excess reducing power, which helps mitigate intracellular redox imbalance. Over time, ethanol was reoxidized to acetate. The produced C2 compounds were upgraded biologically to butyrate (C4) and caproate (C6) using *Clostridium kluyveri*. These are the longest chains derived only by CO_2_ and solar energy reported to date, albeit indirectly. In another study, bias‐free CO_2_‐to‐acetate conversion was achieved by coupling a biophotocathode made of immobilized *S. ovata* on a Si nanowire photocathode with a TiO_2_ photoanode. This design reached 86% acetate selectivity under simulated sunlight, suggesting a distinct ET pathway compared to a previous study that used different materials and an external bias [49].

Polyhydroxybutyrate (PHB) synthesis is the most challenging CO_2_ reduction discussed here due to its long carbon chain and complex ET requirements [127, 128]. PHB is typically produced via MES using *Ralstonia eutropha* and H_2_ as the electron donor, often requiring high applied biases. To improve solar‐driven PHB production, Zhang et al. immobilized *R. eutropha* on a CoP‐Fe_2_O_3_/g‐C_3_N_4_ biophotocathode and coupled it with an oxygen evolution reaction‐catalyst‐decorated Ti mesh [129]. Under illumination and an applied bias of −0.9 V versus Ag/‍AgCl, the system achieved a PHB concentration of 142.20 mg L^−1^ after 7 days. Although this bias is higher than that used for simpler CO_2_ reduction reactions, it is substantially lower than the ~−2.2 V typically required for conventional MES‐based PHB synthesis [130].

Ammonia production heavily relies on fossil fuels, prompting the urgent need to explore greener N_2_ fixation methods. Zhang et al. first coupled N_2_‐fixing bacteria, *Azotobacter vinelandii* (*A. vinelandii*), with a polydopamine‐encapsulated NiO nanosheet photocathode. The polydopamine layer enhanced bacterial loading and interfacial ET [35]. At −0.5 V versus RHE, the biophotocathode (PEC‐biohybrid) achieved an NH_3_ production rate of 4.14 µmol h^−1^ cm^−2^. This represents 2.94‐ and 3.06‐fold improvements over the biased–dark (EC‐biohybrid) and unbiased–light (PC‐biohybrid) controls, respectively (Figure 5f). Subsequently, the same group improved the performance using Cu_2_O@TiO_2_ core–shell nanowire photocathodes. TiO_2_ provided biocompatibility and structural stability, and Cu_2_O enabled broad visible‐light absorption and a highly reducing CB (−0.7 V vs. RHE) [131]. This optimized design increased the NH_3_ production rate to 5.36 µmol h^−1^ cm^−2^ at a reduced bias of −0.3 V versus RHE. This result highlights the importance of band alignment, light harvesting, and biointerface design in solar‐driven biological N_2_ fixation. A summary of the biophotocathode configuration is shown in Table 4.

**TABLE 4:** Summary of biophotocathode configurations (AM1.5G, air mass 1.5 global; ECE, energy conversion efficiency; FE, Faradaic efficiency; IO, inverse opal; NR, not reported; NW, nanowire; OPV, organic photovoltaic; PDA, polydopamine; PHB, polyhydroxybutyrate; STF, solar‐to‐fuel efficiency; STH, solar‐to‐hydrogen efficiency). The reported electrode size is either the physical size or the projected surface area.

Biocatalyst/ET mode	Application	Electrode (size) and working volume (WV)	Light source	Performance	References
Enzyme/MET	H_2_ generation	Biophotocathode: dye‐sensitized NiO/H_2_ase (0.3 cm^2^), Anode: BiVO_4_ (NR), WV: NR	Solar simulator (100 mW cm^−2^, AM1.5G)	Unbiased, FE: 81%, STH: 0.124%	[115]
Enzyme/DET	H_2_ generation	Biophotocathode: OPV/IO‐TiO_2_/H_2_ase+CA (0.19 cm^2^), Anode: BiVO_4_ (0. 25 cm^2^), WV: NR	Solar simulator (100 mW cm^−2^, AM1.5G)	Unbiased, FE: 101%, STH: 0.49%	[121]
Enzyme/DET	H_2_ generation	Biophotocathode: perovskite/IO‐TiO_2_/ H_2_ase (0.28 cm^2^), Anode: BiVO_4_ (NR), WV: NR	Solar simulator (100 mW cm^−2^, AM1.5G)	Unbiased, FE: 82%, STH: 1.1%	[122]
Enzyme/DET	CO_2_ to formate	Biophotocathode: OPV/IO‐TiO_2_/FDH + CA (0.19 cm^2^), Anode: BiVO_4_ (0.25 cm^2^), WV: NR	Solar simulator (100 mW cm^−2^, AM1.5G)	Unbiased, FE: 98%, STF: 0.57%	[121]
Whole cell/MET	CO_2_ to CH_4_	Biophotocathode: p‐InP Pt in *M. barkeri* (1.5 × 2 cm), Anode: n‐TiO_2_ (9 cm^2^), WV: 230 mL	300 W Xe lamp (220 mW cm^−2^, *λ* > 455 nm)	Unbiased, CH_4_ yield: 68.8 nmol (3 days), FE: 74%	[34]
Whole cell/DET	CO_2_ to acetate and ethanol	Biophotocathode: *S. ovata* on CuBi_2_O_4_/MgO (0.25 cm^2^), Anode: Pt (NR), WV: 25 mL	300 W Xe lamp (100 mW cm^−2^, AM1.5G)	Bias: 0 V versus SHE, FE_acetate_: 34%, FE_ethanol_: 35%	[126]
Whole cell/DET	CO_2_ to acetate	Biophotocathode: *S. ovata* on Si NW (NR), Anode: Pt/Ni/TiO_2_/Si NW (NR), WV: NR	Solar simulator (100 mW cm^−2^, AM1.5G)	Unbiased, ECE: 0.38%	[49]
Whole cell/MET	CO_2_ to polyhydroxybutyrate	Biophotocathode: *R. eutropha* on CoP‐Fe_2_O_3_/g‐C_3_N_4_ (4 × 2 cm), Anode: Ir/Ru‐decorated Ti mesh (5 × 2.5 cm), WV: 280 mL	Iodine tungsten lamp (50 W, 16 kLux)	Bias: −0.9 V, PHB concentration (7 days): 142.20 mg L^−1^	[129]
Whole cell/DET	N_2_ fixation	Biophotocathode: *A. vinelandii* on PDA‐encapsulated NiO (1 × 5 cm), Anode: graphite rod (NR), WV: NR	Solar simulator (100 mW cm^−2^, AM1.5G)	Bias: −0.5 V versus RHE, NH_3_ production rate: 4.14 µmol h^−1^ cm^−2^	[35]
Whole cell/DET	N_2_ fixation	Biophotocathode: *A. vinelandii* on Cu_2_O@TiO_2_ nanowire (1 × 1 cm), Anode: graphite rod (NR), WV: 50 mL	Solar simulator (100 mW cm^−2^, AM1.5G)	Bias: −0.3 V versus RHE, NH_3_ production rate: 5.36 µmol h^−1^ cm^−2^	[131]

## 5 Summary and Outlook

This review article discussed the current state of BPEC systems by emphasizing the basic understanding of how photoelectrodes and bioelectrodes work and then systematically classified BPEC into separate and integrated biophotoelectrode architectures. It deeply analyzed the working principles, applications, challenges, and performance of each type, as well as current research trends. Improving BPEC performance requires understanding the interactions among photocatalysts, biocatalysts, electrons, light, and chemical species since these interactions certainly affect device performance. However, such behavior is difficult to study under practical conditions, highlighting the need for a clearer theoretical framework. Advances in artificial intelligence and machine learning may help design suitable materials, but these approaches still depend on a well‐established BPEC theory.

In a BPEC system, photoactive components are required to absorb light and generate reactive charge carriers. This is the crucial factor in determining the performance of a BPEC device. Additionally, a suitable environment for biocatalyst growth must be provided while maintaining physical and chemical stability [132]. Therefore, photocatalysts should be modified to increase light absorption, adjust the band position for the desired application, enhance biocompatibility, and improve stability in biological environments. Some possible approaches currently under examination include heteroatom doping [133], heterostructure design [134], morphology engineering [135], and surface functionalization [136, 137]. In addition, an inevitable issue with solar light utilization is its intermittency, which can disrupt production [138]. Thus, developing a capacitive or dark photocatalyst could help solve this problem by storing excess photogenerated charge over time and releasing it as needed [139, 140].

Biological activities are slow, and their metabolic pathways are designed to allow them to survive rather than help humans solve global issues [30]. Some bacteria may have the desired metabolic mechanism; however, they grow slowly and are difficult to culture. Genetic modification can introduce a metabolic pathway, enzyme, or protein associated with a desired reaction into a fast‐growing biocatalyst [141, 142]. For example, microbial electrochemical treatment of polycyclic aromatic hydrocarbons (PAHs) in sediment is more efficient, affordable, and adaptable than physicochemical treatment [143]. However, the ability of microorganisms for external ET is limited compared to well‐known exoelectrogenic microorganisms [144]. Introducing a gene that expresses the EET‐responsible protein might allow these microorganisms to participate in BPEC for the degradation of PAHs or other persistent organic pollutants.

The biotic–abiotic interface is important for biocatalyst adherence, density, and electrical communication. Some studies regard this factor as the primary determinant of device performance [145, 146, 147]. Modification efforts should focus on the surface properties of photocatalysts, biocatalysts, or both. For example, creating synthetic electron conduits that can directly couple the CymA protein in *S. oneidensis* with the electron acceptor side of Fe or Mn minerals would be a highly desirable and interesting research direction since it could bypass the naturally slow ET process [148]. Additionally, collaborations between material science, nanoscience, microbiology, protein engineering, and computational sciences could be established to develop this concept.

Reactor design is important for ensuring efficient light utilization and microbial survival in anode or cathode cells. This includes the design of electrode placement, reactor size, PEM size, the flow conditions of the reactor fluid, and proper sealing conditions, especially for strict anaerobic bacteria [149]. Many electrode materials for BPEC systems involve high temperatures, hazardous chemicals, and long processing times, consuming a lot of energy and generating a lot of pollutants. Thus, developing a green processing method is important to ensure an environmentally friendly fabrication process. Implementing green manufacturing methods may seem straightforward, but it requires rigorous optimization to produce materials with minimal defects.

Configurations involving dual bioelectrodes with a combination of photoactive materials have rarely been examined such as pairing a biophotoanode with a biophotocathode. A dual biophotoelectrode configuration may perform extremely well in multifunctional applications. In a biophotoanode, photoactive materials boost electron extraction from the biocatalyst, enhancing the oxidation reaction. Conversely, photogenerated electrons from the anode travel to the cathode, where they recombine with photogenerated holes. This leaves high‐energy photogenerated electrons at the cathode to be delivered to the biocatalyst, which can then catalyze high‐energy‐demanding reduction reactions. Therefore, such systems may offer three benefits: fast pollutant biodegradation, electricity production, and highly efficient reduction reactions. These systems could benefit industries that generate liquid waste and CO_2_ such as fertilizer producers. Dual biophotoelectrode systems can treat wastewater, convert CO_2_ into a valuable product, and generate electricity for direct use or storage in a battery simultaneously. Rather than treating each pollutant separately, simultaneous treatment offers a more economic approach that still delivers the benefits of the treatment process. However, several challenges must be addressed to realize the full potential of dual biophotoelectrode configurations, including severe interfacial complexity, reaction kinetics mismatch, biological instability under illumination, mass transport constraints, internal resistance, and poor long‐term durability. Therefore, dedicated research to study and optimize such configurations is highly desired.

Both PEC and BEC systems alone have demonstrated their promising potential for large‐scale applications [150, 151]. Since BPEC is a combination of PEC and BEC, it offers potential for scalability as methods for fabricating large photo‐ or bioelectrodes have been established. The challenges of scalability then lie in how to deposit the biocatalyst on the photoelectrode to achieve highly efficient charge transfer and how to establish effective electrical communication between the photoelectrode and the bioelectrode in a separated electrode architecture. Although most works have focused on lab‐scale devices, one report attempted to fabricate a large‐area biophotoanode made of mesoporous carbon nitride/PS‐II via spray freezing. This was then connected to an enzymatic biocathode made of bilirubin oxidase [152]. The researchers successfully prepared a biophotoanode with an area up to 33 cm^2^ and demonstrated that an array of eight tandem BPEC cells with a total photoactive area of 72 cm^2^ could power low‐power electronics such as LEDs. However, applications are still limited to electricity generation. This suggests that the technological readiness level of BPEC devices is still in its early state. Therefore, more research is needed to improve mechanistic understanding, material selection, bottleneck investigation, and cell design for energy and environmental applications.

Given all the fascinating studies conducted over the past 15 years, it is a significant step to bring these brilliant ideas to society. Using the basic scientific knowledge developed through research, engineers can collaborate to design devices large enough for practical use. One study revealed the potential of biomanufacturing to equalize chemical manufacturing by increasing the number of devices instead of building one mega facility [28]. Bringing a novel technology from the laboratory to the industrial scale is a tedious task that can take decades. Many problems emerge when developing a large system such as defects in material production, challenging operational environments for the biocatalyst, safety considerations, reproducibility, and maintenance. Despite these challenges, BPEC devices have the potential to be used as a sustainable technology to address energy and environmental issues in the future. Fostering collaborations among various disciplines, including science, engineering, and social studies, can help develop this technology and realize a sustainable, advanced society.

## Conflicts of Interest

The authors declare no conflicts of interest.

## Data Availability

The data that support the findings of this study are available from the corresponding author upon reasonable request.
